# A novel peptide targeting Clec9a on dendritic cell for cancer immunotherapy

**DOI:** 10.18632/oncotarget.9624

**Published:** 2016-05-26

**Authors:** Zhongyi Yan, Yahong Wu, Jiangfeng Du, Guodong Li, Shengdian Wang, Wenpeng Cao, Xiuman Zhou, Chunjing Wu, Dan Zhang, Xueli Jing, Yifan Li, Hongfei Wang, Yanfeng Gao, Yuanming Qi

**Affiliations:** ^1^ School of Life Sciences, Zhengzhou University, Zhengzhou, China; ^2^ Institute of Biophysics, Chinese Academy of Sciences, Beijing, China; ^3^ Collaborative Innovation Center of New Drug Research and Safety Evaluation, Zhengzhou, China

**Keywords:** Clec9a, dendritic cells, cancer immunotherapy, peptide

## Abstract

Dendritic cells (DCs) are professional antigen-presenting cells with antigen recognition molecules on the surface. Clec9a is selectively expressed on mouse CD8a^+^ DCs and CD103^+^ DCs subsets, which are functionally similar to human BDCA3^+^ DCs. It is reported that Clec9a is responsible for the antigen cross-presentation of these DC subsets. In the present study, by using phage display technique, we discovered a novel peptide WH, which can selectively bind to mouse Flt3L induced Clec9a^+^ DCs or Clec9a over-expressed HEK-293T cells. Furthermore, by using computer-aided docking model and mutation assay, we observed that Asp^248^ and Trp^250^ are two key residues for Clec9a to bind with peptide WH. When coupled with OVA_257-264_ epitope, peptide WH can significantly enhance the ability of Clec9a^+^ DCs to activate OVA-specific CD8^+^ T cells, which elicit strong ability to secret IFN-γ, express perforin and granzyme B mRNA. In B16-OVA lung metastasis mouse model, WH-OVA_257-264_ fusion peptide can also enhance the activation of CD8^+^ T cells and decrease the lung metastasis loci. All these results suggested that peptide WH could be considered as an antigen delivery carrier targeting Clec9a^+^ DCs for cancer immunotherapy.

## INTRODUCTION

Dendritic cells are the professional antigen-presenting cells (APCs) which play important roles in activation of naïve CD8^+^ T cell in response to foreign antigens or dead cell derived antigens [1, 2]. In mice, CD8a^+^ DCs (functionally similar to the BDCA3^+^ DCs in human) are the major subset for antigen cross-presentation which responsible for the activation of anti-tumor CD8^+^ T cell response [3, 4]. Therefore, it raises the possibility to design anti-tumor vaccines targeting this subset of DCs. Some specific C-type lectin members expressed on CD8a^+^ DCs, such as Clec9a (also named as DNGR-1) [3, 5], DEC205[6, 7] and Dectin-1[8, 9], are reported to be responsible for antigen uptake. Among them, Clec9a is uniquely expressed on mouse CD8a^+^DC, CD103^+^DC and also some pDC [3, 5]. Correspondingly, Clec9a is also selectively expressed on human BDCA3^+^ DC [10–12]. Therefore, Clec9a is a very promising target to the DC vaccines for anti-tumor use.

Antigen targeting DCs via Clec9a could strongly enhance anti-tumor immunity [3, 4]. Other evidence also showed that antigen targeting Clec9a can enhance immune responses of CD4^+^ T cell, CD8^+^ T cell and B cell [5, 13, 14]. Interestingly, even in the absence of adjuvant, antigen targeting Clec9a can also promote potent humoral immunity and drive long effective development of follicular helper T cells, different from that of antigen targeting DEC205[13]. Further study indicated that the Clec9a can specifically recognize F-actin, a main component of the cellular cytoskeleton exposed by necrotic cells, and initiate cross priming of DCs to CD8^+^ T cell response against dead cell-associated antigens [15–17]. These results suggested that Clec9a is a DC-restricted marker sensing damaged cells and antigens, thus targeting Clec9a^+^ DC can promote humoral and cellular immunity. Actually, Clec9a antibody conjugated with antigen could enhance the cross priming of DC cells, and simulate cytotoxic T lymphocytes (CTLs) response to elicit anti-tumor effects [3, 18].

Considering that small peptides are more convenient to be conjugated to CTL epitopes by solid phase peptide synthesis methods, we proposed that small peptides could also be used as antigen carrier targeting Clec9a. To achieve this, we screened the Clec9a binding peptides by using phage display library. We found that a novel peptide WH can strongly bind to Clec9a^+^ DCs or Clec9a^+^ over-expressed HEK-293 cells. By computer aided docking and mutation study, we further identified the key residues of Clec9a contact with peptide WH. We coupled peptide WH with model antigen peptide OVA_257-264_, and studied the cross priming of DCs to stimulate antigen specific CD8+ T cell response. In the B16-OVA lung metastasis mouse model, the CTL induction capacity and anti-tumor activity of the fusion peptide WH-OVA_257-264_ were also investigated.

## RESULTS

### Clec9a binding peptides screening and validating

**Figure 1:**
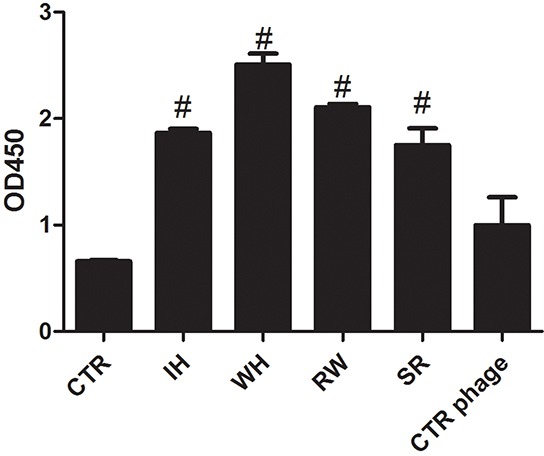
ELISA of the selected phage clones toward Clec9a-CTLD protein 10μg Clec9a-CTLD protein was coated into the ELISA plate at 4°C overnight. The next day, the plate was washed 3 times with TBS buffer containing 0.05% Tween 20 and blocked with 5% BSA buffer. 1 × 10^7^ phages were added for 2 hours at room temperature. The anti-M13 protein with HRP and 1×TMB buffer were used. Finally, 50 μL 1mM H_3_PO_4_ stop buffer was added and plate was read at 450nm. #: twice higher than control group (CTR, without phage). CTR phage: phage that do not have random peptide sequence inserted.

**Figure 2:**
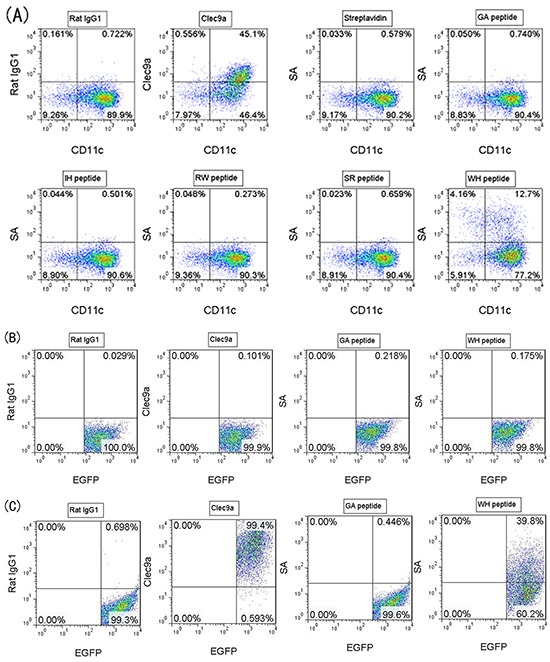
Binding of peptides toward Clec9a^+^ FL-DCs or transfected HEK-293T cells Each biotinylated peptide was incubated with FL-DCs (or transfected HEK-293T cells) at 4°C for 30min, washed 3 times with FACS buffer containing 2mM EDTA, and then streptavidin-PE (SA) was added to stain the peptide binding to FL-DCs (or transfected HEK-293T cells) at 4°C for another 30min. After Washing twice, the cells were analyzed by flow cytometry. Peptide GA was used as peptide control, and Rat IgG1 was used as isotype control. **A.** Clec9a was expressed on DCs induced by 200ng/mL Flt3L (FL-DCs). Peptide WH but not other peptides can bind to FL-DCs. **B.** HEK-293T was transfected with the pIRES2-EGFP plasmid (mock vector) by using calcium phosphate cell transfection method. Peptide WH can not bind to the mock transfectant (pIRES2-EGFP) Clec9a^−^ HEK-293T cells. **C.** HEK-293T was transfected with the pIRES2-EGFP-mClec9a plasmid by using calcium phosphate cell transfection method. Peptide WH can bind to Clec9a^+^ HEK-293T cells.

It was reported that the C-type lectin-like domain (CTLD) of Clec9a was important to the antigen recognition and endocytosis. Therefore, bio-panning was performed by using 12-mer peptide phage library targeting mouse Clec9a-CTLD fragment. After five rounds of bio-panning, the exceptional enrichment of phage binders was obtained. After sequencing of the random selected 40 phage clones, four peptides were identified (Table 1). All the four corresponding phage clones can bind to the Clec9a-CTLD protein in the ELISA assay (Figure 1), especially the phages with the sequence of peptide WH. To investigate whether these four peptides could bind to Clec9a^+^ DCs, bone marrow cells induced by Flt3L (FL-DCs) were used. As shown in Figure 2A, Clec9a is highly expressed on Flt3L induced DCs, and only peptide WH could bind to FL-DCs. To further verify the binding ability of peptide WH to Clec9a, mClec9a-EGFP was over-expressed in HEK-293T cells. As shown in Figure 2B, peptide WH can not bind to the mock plasmid transfected HEK-293T cells. As expected, peptide WH but not the control peptide (GA) can bind to Clec9a^+^ HEK-293T cells (Figure 2C). The results suggested that peptide WH could selectively bind to Clec9a.

**Table 1:** Clone frequency and peptide sequences of the selected phages

Phage	Peptide sequence	Phage frequency
IH	IKTGPASLQNPH	20/40
WH	WPRFHSSVFHTH	4/40
RW	RPHRNGGRDQTW	2/40
SR	SAHNPSSLTGSR	2/40

### The interaction between peptide WH and the Clec9a-CTLD fragment

*In silico* approaches were applied to elucidate peptide binding model of Clec9a, of which the 3D structure was retrieved from protein data bank, and optimized by adding exclusive hydrogen atoms and the missing atoms to make the protein in protonated state. The 3D structure of peptide WH was created by using PEP-FOLD. Next, the 3D structure of peptide WH was unbiased docked to the optimized structure of Clec9a (UniProtKB: Q8BRU4) by using ZDOCK, which in turn created 50 most possible docking poses (WH/Clec9a complexes). The docked poses were analyzed and clustered into 2 groups: S1 and S2 (Supplementary Figure S1). The residues of Clec9a contact with peptide WH was listed in Supplementary Table S1.

**Figure 3:**
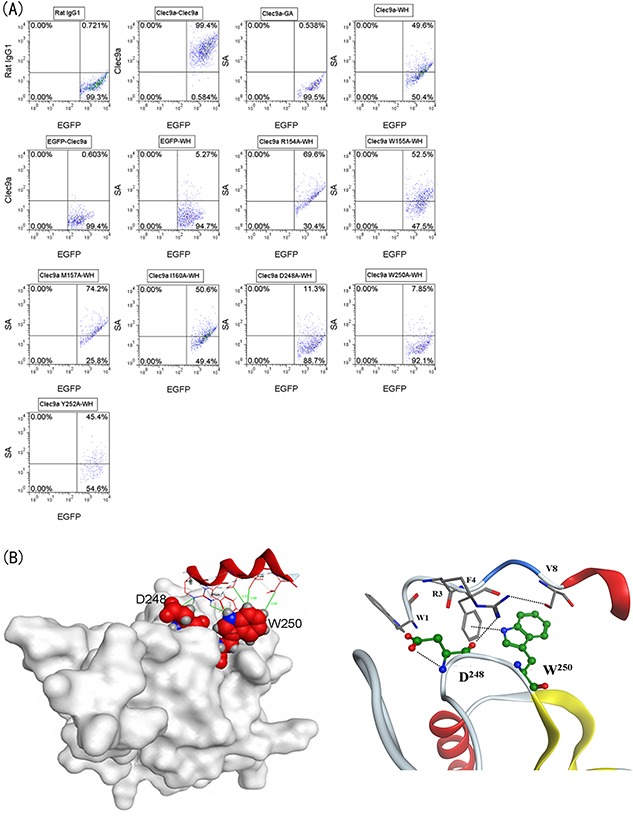
Binding of peptide WH toward Clec9a mutants and the docking model PEP-FOLD was used to mimic the structure of peptide WH, and then these structures were docked to mouse Clec9a crystal structure for predicting the key binding residues of Clec9a. **A.** Seven Clec9a single amino acid mutation plasmids were transfected into HEK-293T cells. The staining procedure was same as that described in Figure 2. **B.** The left figure showed the docking model of peptide WH with mClec9a. The right figure showed that the key interaction forces less than 4.5Å of peptide WH bind to D^248^ or W^250^ of Clec9a.

The output of the *in silico* prediction guided side-directed mutagenesis experiments, which was retrospectively used to verify the quality of the peptide binding model. The docking poses suggested that seven residues of Clec9a may play crucial role in peptide binding. Therefore, mutagenesis experiments were conducted and seven Clec9a mutants were expressed on HEK-293T cells. As shown in Figure 3A, when the residue Asp^248^ (D^248^) or Trp^250^ (W^250^) of Clec9a was mutated to alanine, the binding activity of peptide WH was greatly impaired. Echoing to the peptide WH/Clec9a docking model, the residue W^250^ can interact with five residues of peptide WH (Arg^3^, Phe^4^, Ser^7^, Val^8^ and Thr^11^), and D^248^ can interact with Arg^3^ and Phe^4^ of peptide WH (Figure 3B). The results also showed that Arg^3^ and Phe^4^ of peptide WH are the major residues interact with D^248^ and W^250^ of Clec9a, with less than 4.5Å distance (Figure 3B). These results further confirmed that peptide WH could bind to Clec9a specifically.

### Peptide WH enhances OVA_257-264_ cross presentation by Clec9a^+^ DCs

**Figure 4:**
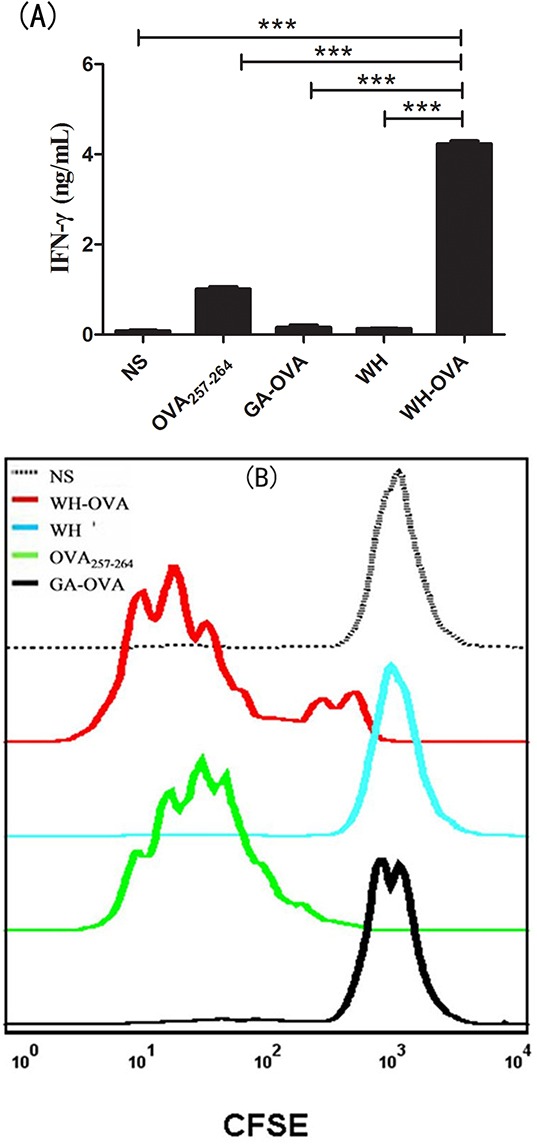

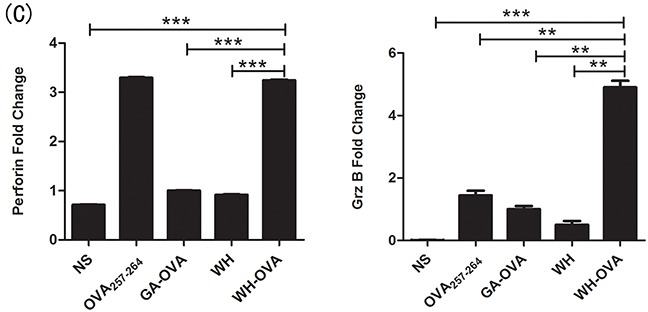
The effects of peptide WH on the cross-presentation of OVA_257-264_ by FL-DCs *in vitro* FL-DCs were pulsed with different peptides (10μg/mL) or normal saline at 4°C for 1.5 h, and then washed 3 times with medium. Isolated lymph node CD8^+^ T cells from OT-1 mice were labeled with 2μM CFSE for 10min at room temperature, and washed 3 times with medium. The 1×10^5^ CFSE stained CD8^+^ T cells were incubated with different peptides pulsed 1×10^4^ FL-DCs for 72 h. **A.** The IFN-γ of the supernatant determined by ELISA. **B.** Proliferation of CD8^+^ T cells stimulated by peptide pulsed FL-DCs. **C.** The perforin and granzyme B (Grz B) mRNA expression fold change compared to the control peptide GA-OVA_257-264_ group. Data are presented as means ± SEM, **P<0.01, ***P<0.001. Data are representative of three experiments.

Since Clec9a is highly expressed on Flt3L induced DCs, FL-DCs are always considered as the surrogate of CD8a^+^DCs. To see whether peptide WH could enhance the cross-presentation of OVA_257-264_ antigen, the fusion peptide WH-OVA_257-264_ and GA-OVA_257-264_ control were synthesized. As shown in Figure 4, after incubation with WH-OVA_257-264_, FL-DCs could strongly stimulate the IFN-γ release from OT-1 CD8^+^ T cells. This activation effects are more potent than that of the control peptide GA-OVA_257-264_ or OVA_257-264_ alone. WH-OVA_257-264_ treated FL-DCs can also stimulate the proliferation of OT-1 cells. We also detected the mRNA expression of CTL killing markers perforin and granzyme B by qRT-PCR. The mRNA expression of granzyme B of WH-OVA_257-264_ group is about 5-fold higher than that of GA-OVA_257-264_. These results suggested that the peptide WH can deliver antigen to Clec9a^+^ DCs, enhance the antigen cross-presentation, and thus promote T cell activation.

### Peptide WH enhances OVA_257-264_ antigen specific CTL cross priming *in vivo*

**Figure 5:**
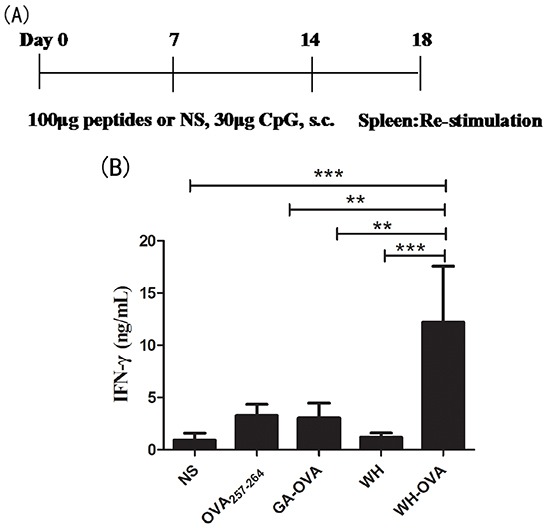

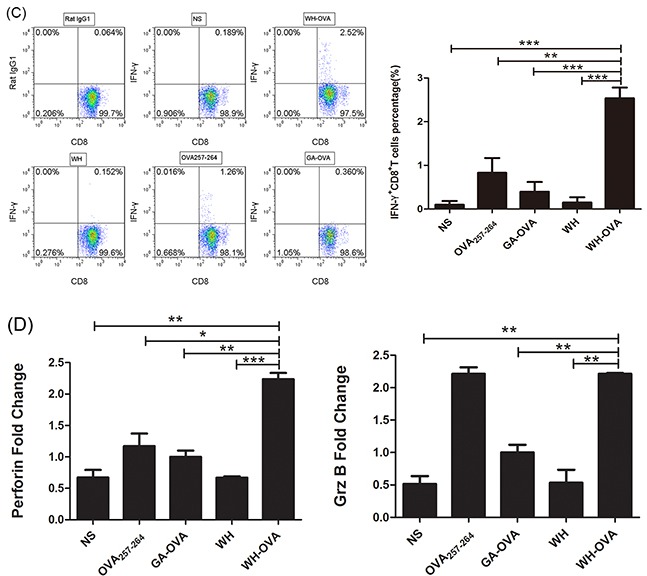
The effects of peptide WH on the cross priming of OVA_257-264_ specific CD8^+^ T cells in naïve C57Bl/6J mice 100μg WH-OVA/OVA_257-264_/WH/GA-OVA or normal saline (NS) was injected s.c. with CpG ODN 1826 (30μg) weekly for three times, then the splenocytes were isolated and re-stimulated by OVA_257-264_ (10μg/mL) for 5 days. **A.** The scheme of the immunization and process. **B.** Supernatant IFN-γ determined by ELISA. **C.** The frequencies of IFN-γ released by OVA_257-264_ specific CD8^+^ T cells analyzed by flow cytometry intracellular staining. The histogram showed the statistical results. **D.** The perforin and granzyme B mRNA expression fold change compared to the control peptide GA-OVA_257-264_ group. Data are presented as means ± SEM (n=5), *P<0.05, ** *P*<0.01, *** *P*<0.001. Data are representative of two experiments.

Since peptide WH can enhance antigen cross priming *in vitro*, we wonder if this peptide can also act as a vaccine carrier and promote the antigen-specific CTLs induction *in vivo*. After immunization of the mice with peptide WH-OVA_257-264_ or controls for three times, the splenocytes were re-stimulated with OVA_257-264_ to detect the CTL response via IFN-γ secretion and CTL killing markers expression, such as perforin and granzyme B. As shown in Figure 5, peptide WH-OVA_257-264_ can strongly stimulate supernatant and intracellular IFN-γ secretion compared with the control groups. In the intracellular IFN-γ staining assay, WH-OVA_257-264_ significantly enhance the induction of OVA_257-264_ specific CD8^+^ T cell response. Also, the mRNA expression of perforin and granzyme B was both increased. All these data indicated that peptide WH can enhance the cross presentation of antigen OVA_257-264_ *in vivo* and has the potential to be used as a vaccine carrier.

### Peptide WH enhances antitumor effects of OVA_257-264_

**Figure 6:**
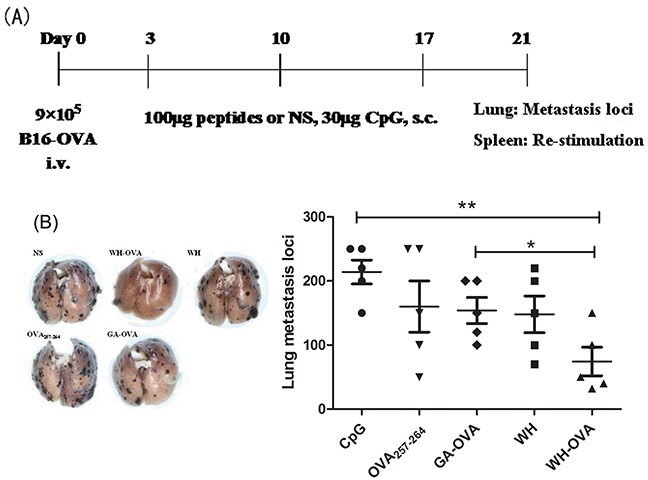

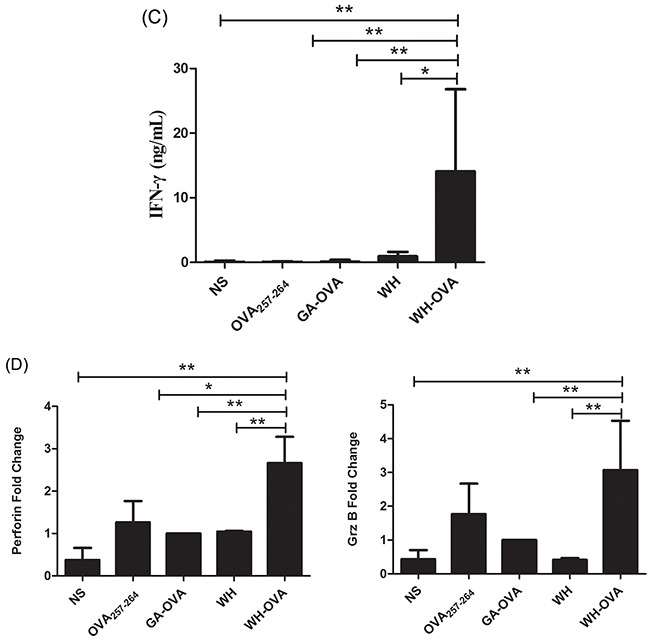
The antitumor effects of peptide WH-OVA_257-264_ in B16-OVA melanoma lung metastasis model 9×10^5^ B16-OVA melanoma cells were injected intravenously in C57Bl/6J mice to establish the lung metastasis model. 3 days later, 100μg WH-OVA/OVA_257-264_/WH/GA-OVA or NS was injected s.c. with CpG ODN 1826 (30μg) weekly for three times, then the lungs were processed to count the metastasis loci, and the splenocytes were isolated and re-stimulated by OVA_257-264_ (10μg/mL) for 5 days. **A.** The scheme of tumor model, immunotherapy and *ex vivo* analysis. **B.** The effects of peptide WH-OVA_257-264_ on the number of lung metastasis loci. **C.** Supernatant IFN-γ determined by ELISA. **D.** The perforin and granzyme B mRNA expression fold change. Data are presented as means ± SEM (n=5), *P<0.05, **P<0.01. Data are representative of two experiments.

To investigate whether the fusion peptide WH-OVA_257-264_ could be used as a vaccine for the immunotherapy of cancer metastasis, the lung metastasis model of B16-OVA was established. As shown in Figure 6, the number of lung metastasis loci was significantly decreased in the group treated with WH-OVA_257-264_ compared to that of control group. In the tumor bearing mice, WH-OVA_257-264_ could strongly stimulate IFN-γ production of the splenocytes than that of control groups (GA-OVA, OVA_257-264_ or WH peptide). Furthermore, WH-OVA_257-264_ treatment can induce CTL killing markers perforin and granzyme B mRNA expression. Overall, the results suggested that peptide WH as a promising vaccine carrier can stimulate antigen specific CTL cross-priming and enhance anti-tumor immunotherapy.

## DISCUSSION

Dendritic cells based vaccines are one of the most promising candidates for cancer immunotherapy [19, 20]. Application of tumor associated antigens pulsed DC vaccines has achieved some success [21]. But most of these vaccines could not induce strong T cell response. One reason is that these antigens can not be well uptaken and presented by DCs. To overcome this, researchers discovered a series of potential molecules on the surface of DCs, which could help DCs to recognize, uptake and process antigens. Among these molecules, C-type lectin members, such as DEC205[22–25], Clec9a [3–5, 15, 26] and Dectin-1 [8, 9, 27, 28] are extensively studied. Clec9a is selectively expressed on CD8a^+^ DCs (mouse) and BDCA3^+^ DC (human), the subsets with the most strongest ability to the cross-presentation of antigens. It was reported that Clec9a antibody conjugated with antigens targeting Clec9a^+^ DC could promote the antigen cross presentation and enhance antitumor immunotherapy [3, 5, 13, 18, 29]. To take the advantage of small peptides which could be conveniently synthesized with epitopes or fused with protein antigens, we performed the widely used phage display method to screen Clec9a binding peptides. Then we tested its ability to act as an antigen carrier targeting Clec9a^+^ DCs both *in vitro* and in tumor bearing mice.

The newly discovered peptide WH can bind to Flt3L induced DCs or mClec9a^+^ HEK-293T cells. This binding activity is correlated to the expression of Clec9a. Moreover, peptide WH can not binding the tested murine cell lines, such as B16, B16-F10, S180, CT26, H22, RAW264.7 and NIH3T3 (data not shown). We also conducted mutagenesis experiment and identified that the residues D^248^ and W^250^ were crucial for peptide WH binding. The results were further confirmed by unbiased docking study through PEP-FOLD and ZDOCK. The results were consistent to the previous report that mutation of W^250^ of Clec9a significantly impaired F-actin recognition [16]. All these results suggested that peptide WH could selectively and specifically bind to Clec9a.

WH-OVA_257-264_ treated FL-DCs can significantly stimulate CD8^+^ T cells to release IFN-γ compared to that of GA-OVA_257-264_ or OVA_257-264_ alone. Also, CD8^+^ T cells simulated by WH-OVA_257-264_ treated FL-DCs exhibit good proliferation, express high level of perforin and granzyme B. When immunizing WH-OVA_257-264_ in mice, antigen specific CD8^+^ T cell response could be induced, which indicated that peptide WH could enhance the cross presentation of OVA_257-264_ epitope *in vivo*.

Then, by using lung metastasis tumor bearing mice model, we found that peptide WH could greatly enhance the OVA_257-264_ antigen specific T cell response and decrease the lung metastasis loci. Our results suggested that peptide WH could be very powerful to be used as antigen carrier to help the anti-tumor immunotherapy of DC vaccines. Compared to the anti-Clec9a antibody, this short peptide is very convenient to be synthesized and conjugated to the antigen epitope. In our lab we are also trying to combine this strategy with checkpoint blockade inhibitors to further enhance the antitumor effects [30].

In conclusion, a novel peptide targeting Clec9a was identified by phage display bio-panning. Peptide WH could bind to Clec9a^+^ DCs selectively, and enhance the antigen cross presentation, further stimulate the antigen specific CTL response both *in vitro* and *in vivo*. This strategy could be very effective in cancer immunotherapy.

## MATERIALS AND METHODS

### Mice

C57Bl/6J (Beijing HFK Bioscience Co., China) and OT-1 mice were bred at Zhengzhou University in specific pathogen-free facility. All animal experiments were performed in accordance with national and institutional guidelines for animal care, and were approved by Ethics Committee of Zhengzhou University.

### Cells

Mouse B16 melanoma cell line was obtained from the Type Culture Collection of the Chinese Academy of Science (Shanghai, China). OVA antigen transgenic cell line B16-OVA was maintained in Prof. Shengdian Wang's lab. B16 and B16-OVA cells were cultured in RPMI 1640. HEK-293T cells were cultured in DMEM. For FL-DC induction, mouse BMDCs were generated by culturing bone marrow cells in RPMI 1640 supplemented with 200ng/mL Flt3L (eBioscience, USA) for 10~12 days with semi-medium changing every 4 days. Splenocytes were prepared from C57BL/6J by removing red blood cells (RBCs, 0.168M NH_4_ Cl; 5 minutes on ice). CD8a^+^ T cells of OT-1 mice were isolated from lymph nodes by using EasySep™ Mouse CD8^+^ T Cell Isolation Kit (Stemcell Technologies). All the culture medium was supplemented with 10% fetal calf serum (BI), penicillin, streptomycin, glutamine, and 2-macraptoethanol (Life technology), and cells were cultured at 37°C in 5% CO_2_.

### Protein expression and mutation

The 3×FLAG-mClec9a-CTLD plasmid was a kind gift from Professor Caetano Reis e Sousa and Kathryn Snelgrove [15]. The mClec9a-CTLD fragment was expressed by HEK-293T cells, and then purified by anti-FLAG (M2) beads (Sigma) and size-exclusion chromatography pre-packed Superdex 200 (GE). In the cellular binding experiments, Clec9a gene was cloned from splenocyte cDNA of C57Bl/6J mice and constructed into pIRES2-EGFP vector (Biofeng, China). The forward primer: 5′-CCCTCGAGATGCATGCGGAAGAAATATATACCTC-3′; reverse primer: 5′-CGGAATTCTCAGATGCAGGATCCAAATGC-3′. The recombinant plasmid was expressed in HEK-293T cells for peptides binding experiments. For peptide WH/Clec9a interaction study, PEP-FOLD was used to predict the peptide 3D structures of the peptide, and then the top 50 peptide WH structures were used to dock with the optimized Clec9a unbiasly by using ZDOCK. Most of the docked poses located in S1 (R^154^, E^156^, M^157^, N^159^, I^160^, S^161^, K^163^, S^164^, K^167^, C^247^, D^248^ and W^250^) or S2 (E^153^, W^155^, K^191^, N^194^, S^249^, and Y^252^) area of Clec9a. Top 10 poses of WH/Clec9a complexes were selected to statistically calculate the occurrence of residues in binding of Clec9a (Supplementary Table S1). The main residues of Clec9a for the interaction with peptide WH were R^154^, W^155^, M^157^, I^160^, D^248^, W^250^ and Y^252^. Each of these residues was mutated to alanine by using quick change method by using the pIRES2-mClec9a plasmid as template. The primers were shown in Supplementary Table S2.

### Bio-panning of mClec9a protein binding phages

The Ph.D.™ −12 Phage Display Peptide Library Kit was purchased from New England Biolabs (NEB, Beijing, China). The panning experiment was conducted according to the manufacturer's instructions. Briefly, 100 μg/mL mClec9a protein was coated onto a 96-well plate overnight at 4°C with gentle agitation in a humidified container in 0.1M NaHCO_3_ pH8.6 solution. After washing with Tris-buffered saline (TBS containing 0.1% Tween 20), 0.1M NaHCO_3_ pH8.6 blocking buffer containing 5mg/mL BSA was added for 3h incubation at 4°C. After washing with TBST, 10^13^pfu phages were added for 1h incubation at 25°C with gentle shaking. Then the unbound phages were removed. 200μL 0.2M Gly-HCl (pH2.2 with 0.1% BSA) was used to harvest the binding phages. After five rounds bio-panning, 40 phage clones were selected randomly and sequenced.

### Peptide synthesis

Peptides were synthesized in our lab by using standard solid phase Fmoc synthesis strategy and purified to more than 95% purity by reverse phase-high performance liquid chromatography (RP-HPLC). Their molecular weights were assessed by mass spectrometry [31]. For flow cytometry (FCM) binding assay, peptides were added with *GGGK-biotin* at carboxyl terminal. Peptide sequences were as follows: WPRFHSSVFHTH*GGGK-biotin* (WH-biotin); WPRFHSSVFHTH*GGG*SIINFEKL (WH-OVA_257-264_); GAGAAGGAGGGG*GGGK-biotin* (GA-biotin, control peptide) [32]; GAGAAGGAGGGG*GGG*SIINFEKL(GA-OVA_257-264_); SIINFEKL(OVA_257-264_); WPRFHSSVFHTH (WH).

### Peptide binding assay by flow cytometry

In brief, 5 × 10^5^ FL-DCs or HEK-293T cells transfected with pIRES2-mClec9a were used. For all flow cytometry assays, the cells suspension was incubated with rat serum to block Fc receptors, and stained with corresponding antibody or biotin-peptides at 4°C for 30 min, and then analyzed by flow cytometry after washing twice.

### *In vitro* cross presentation of OVA_257-264_ epitope by DC

*In vitro* peptide stimulation T cell assays were performed using a modification of a previously described protocol [32]. In brief, 10μg/mL peptides (peptide-OVA_257-264_, OVA_257-264_, WH or NS) were incubated with FL-DC Day12 for 1.5 h at 4°C, by which the end time the peptide-loaded FL-DCs were washed three times. Meanwhile, OT-I CD8^+^ T cells were isolated using EasySep^TM^ CD8^+^ T cell negative selection kit (Stemcell Technologies). Subsequently, the CD8^+^ T cells were seeded at 10^5^ cells per well at round-bottom 96-well plates for 30 min. The peptide-loaded FL-DCs were then gently added at 10^4^ cells per well to the final volume of 200μL. After incubation for 72 h, Supernatants were assayed for IFN-γ by ELISA. Perforin and granzyme mRNA expression were tested by qRT-PCR. The perforin forward and reverse primers are 5′-AGCACAAGTTCGTGCCA GG-3′ and 5′-GCGTCTCTCATTAGGGAGTTTT T-3′, respectively. The granzyme B forward and reverse primers are 5′-CCACTCTCGACCCTACATGG-3′ and 5′- GGCCCCCAAAGTGACATTTATT-3′, respectively. The β-actin forward and reverse primers are 5′-GTGGCATCCATGAAACT ACAT-3′ and 5′- GGCATAGAGGTCTTTACGG-3′, respectively. For T cell proliferation assay, the CD8^+^ T cells were stained with 2μM CFSE. Three days later, CD8^+^ T cell proliferation was determined by CFSE dilution.

### CTL priming

To test the peptide-OVA_257-264_ stimulation of CTL response, the peptide-OVA_257-264_, WH or OVA_257-264_ (100μg per mouse) were injected s.c. in the back of C57BL/6 mouse with 30μg CpG ODN1826, as an adjuvant, for three times every 7 days internal. Five days after the last injection, *ex vivo* CTL assays were performed as described previously [3]. In brief, for re-stimulation of CTLs *in vitro*, splenocytes were incubated with 10μg/mL OVA_257-264_ peptide for 5 days. The supernatant IFN-γ was measured by ELISA. The perforin and granzyme B mRNA expression were tested by qRT-PCR. Meanwhile, the IFN-γ release of CTLs were also determined by intracellular staining. Briefly, the re-stimulated splenocytes were added protein transport inhibitor cocktail for 7 hours, then those cells were stained with anti-CD3-PerCP-eFlour 710 (17A2) and anti-CD8-PE (53-6.7) for 30min at 4°C. After the cells were fixed by fixation buffer (eBioscience) for another 30 min at room temperature and were washed twice by permeabilization wash buffer (eBioscience). Anti-IFN-γ -APC (XMG1.2) was added into those cells for intracellular staining for 30min at 4°C. After being washed twice by permeabilization wash buffer, IFN-γ released by CD8^+^ T cells was analyzed by flow cytometry.

### Tumor model

The mice were received 9 × 10^5^ B16-OVA cells i.v. Three days later, these mice were injected weekly for 3 times with 100μg peptide-OVA_257-264_/peptide WH/OVA_257-264_, 30μg/mouse CpG ODN 1826 as adjuvant. After 5 days of the last injection, these mice were sacrificed. Tumor burden was assessed by counting lung foci. Meanwhile, the splenocytes were pulsed with 10μg/mL OVA_257-264_ peptide for 5 days. The supernatant IFN-γ was determined by ELISA. Also the CTL response were monitored as described above.

### ELISA for detection supernatant IFN-γ

The experiment was according to the mouse IFN-gamma ELISA Ready SET-Go kit (eBioscience). Briefly, ELISA plate (Costar) was coated with 100 μL/well of IFN-γ capture antibody overnight at 4°C. Next day, aspirate wells and wash 3 times with washing buffer (PBS pH7.2 with 0.05% Tween 20), then block with ELISA/ELISPOT buffer 1hour at room temperature. Serially diluted supernatant samples or IFN-γ standard were added into wells and incubated for 2 hours at room temperature. Bound biotinylated IFN-γ were detected using avidin-HRP and 1×TMB solution. At the end time, 50μL/well Stop Solution was add and the plate was read at 450nm.

### Perforin and granzyme B mRNA expression by quantitative RT-PCR

Total mRNA from cells were extracted by total mRNA kit (BeiBei Biology, China). Total mRNA reversely transcripted into cDNA using the Revert Aid cDNA synthesis kit (Themo Scientific, USA). PCR reactions were performed as following, 5 min at 65°C, 5 min at 4°C, followed by 2 two-step cycles of 60min at 42°C and 5 min at 70°C. Real-time PCR was performed by the relative standard curve method on Roche LightCycler 480 with LightCycler480 qRT-PCR mix according to the manufacturers' instructions. The PCR reaction were performed as following, 5 min at 94°C for pre-incubation, the detecting fluorescence was 15s at 94°C, 1min at 60°C for 45 cycles. At last the melting curve was 73°C to 95°C, reading every 0.3°C.

### Statistical analysis

Statistical analysis was conducted with 1-tailed Student's t test for differences between groups. The data were shown as means ± SEM unless otherwise stated. *P<0.05, **P<0.01, and ***P<0.001.

## SUPPLEMENTARY FIGURE AND TABLES


