# Supplementary material for: A novel peptide targeting Clec9a on dendritic cell for cancer immunotherapy
Source: Oncotarget. 2016 May 26;7(26):40437–50. doi: 10.18632/oncotarget.9624 (PMC5130018; doi:10.18632/oncotarget.9624)
Supplement: Supplementary file 1 [file oncotarget-07-40437-s001.pdf]

A novel peptide targeting Clec9a on dendritic cell for cancer immunotherapy

SUPPLEMENTARY FIGURE AND TABLES

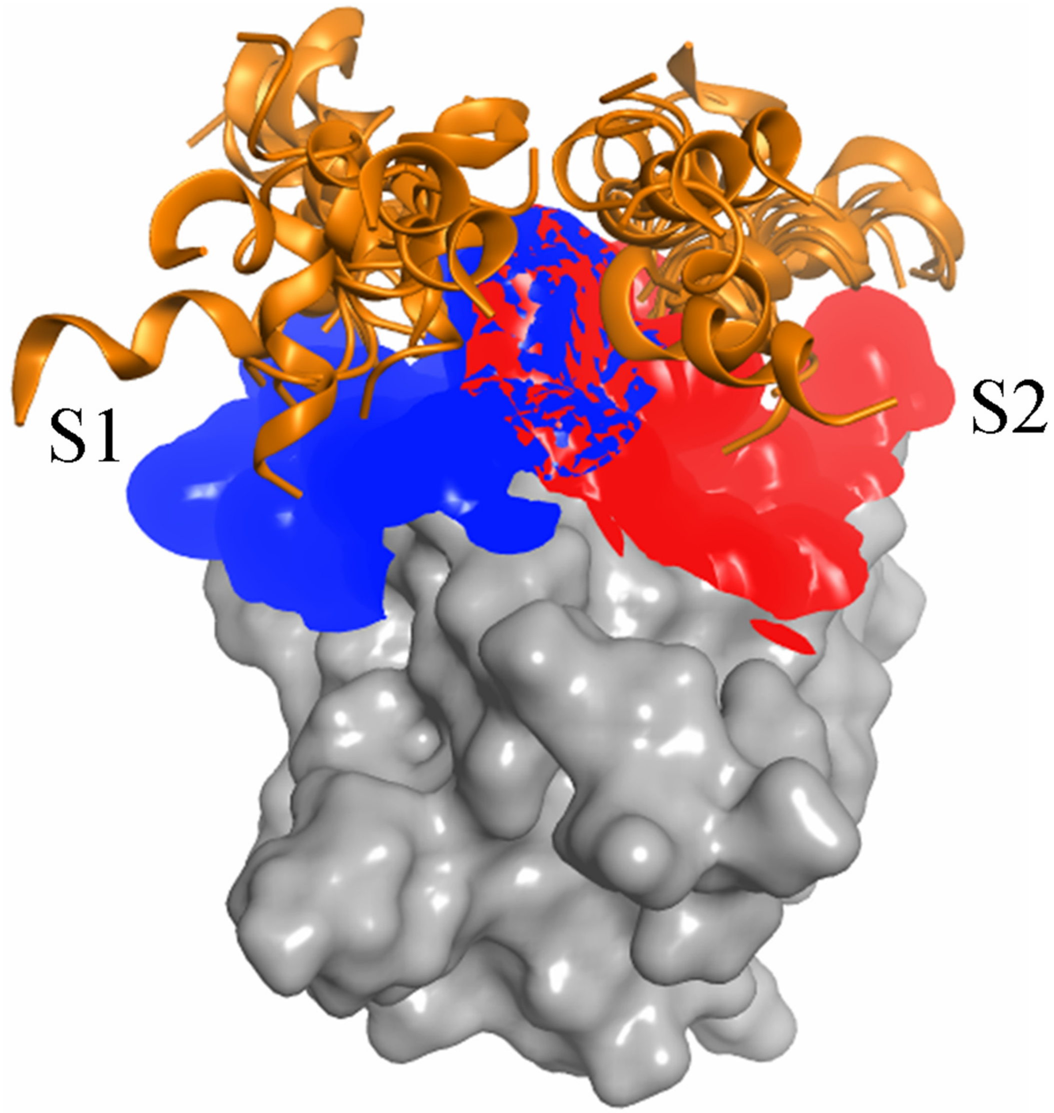

Supplementary Figure S1: The peptide WH-mClec9a docking model. PEP-FOLD was used to predict the 3D structure and conformation of peptide WH. Next, the 3D structure of peptide WH was unbiased docked to the crystal structure of mClec9a (UniProtKB: Q8BRU4) by using ZDOCK, which then created 50 most possible docking poses (peptide WH-mClec9a complexes). The output docked poses were analyzed and could be clustered into 2 groups: S1 (the blue) and S2 (the red).

Supplementary Table S1: The frequency and docking sites of peptide WH interacts with mClec9a

Clec9a residue	The frequency and docking site of peptide WH
E^{153}	S2-5
R^{154}	S1-8, S2-5
W^{155}	S1-4, S2-18
E^{156}	S1-5
M^{157}	S1-11
N^{159}	S1-2
I^{160}	S1-12
S^{161}	S1-1
K^{163}	S1-1
S^{164}	S1-1
K^{167}	S1-5
K^{191}	S2-4
N^{194}	S2-3
C^{247}	S1-2
D^{248}	S1-6
S^{249}	S1-1, S2-3
W^{250}	S1-16, S2-13
Y^{252}	S2-8

Supplementary Table S2: Clec9a single amino acid mutation primer

mutation	Forward primer (5' to 3')	Reverse primer (5' to 3')
R154A	GTTGTTACTATGTCTTTGAAGC<br>CTGGGAAATGTGGAACATCAG	CTGATGTTCCACATTTCCCAG<br>GCTTCAAAGACATAGTAACAAC
W155A	GTTGTTACTATGTCTTTGAACGCG<br>CGGAAATGTGGAACATCAGTAAG	CTTACTGATGTTCCACATTTCCGC<br>GCGTTCAAAGACATAGTAACAAC
M157A	CTATGTCTTTGAACGCTGGGAAG<br>CGTGGAACATCAGTAAGAAGAGC	GCTCTTCTTACTGATGTTCCACG<br>CTTCCCAGCGTTCAAAGACATAG
I160A	GAACGCTGGGAAATGTGGAACG<br>CCAGTAAGAAGAGCTGTTTAAAAG	CTTTTAAACAGCTCTTCTTACTG<br>GCGTTCCACATTTCCCAGCGTTC
D248A	CTACTCTCATCTCAGATAAGTGCGC<br>TAGCTGGAAATATTTTATCTGTG	CACAGATAAAATATTTCCAGCTA<br>GCGCACTTATCTGAGATGAGAGTAG
W250A	CATCTCAGATAAGTGCGATAGCGCG<br>AAATATTTTATCTGTGAGAAG	CTTCTCACAGATAAAATATTTCGC<br>GCTATCGCACTTATCTGAGATG
Y252A	CAGATAAGTGCGATAGCTGGAAA<br>GCTTTTATCTGTGAGAAGAAGGC	GCCTTCTTCTCACAGATAAAAGCTT<br>TCCAGCTATCGCACTTATCTG
